# The Effect of Non-immersive Virtual Reality Exergames Versus Band Stretching on Cardiovascular and Cerebral Hemodynamic Response: A Functional Near-Infrared Spectroscopy Study

**DOI:** 10.3389/fnhum.2022.902757

**Published:** 2022-07-12

**Authors:** Yuxin Zheng, Tingting You, Rongwei Du, Jiahui Zhang, Tingting Peng, Junjie Liang, Biyi Zhao, Haining Ou, Yongchun Jiang, Huiping Feng, Anniwaer Yilifate, Qiang Lin

**Affiliations:** ^1^Department of Rehabilitation Medicine, The Fifth Affiliated Hospital of Guangzhou Medical University, Guangzhou, China; ^2^Department of Rehabilitation Medicine, The Fifth College of Guangzhou Medical University, Guangzhou, China; ^3^Department of Clinical Nutrition, The Fifth Affiliated Hospital of Guangzhou Medical University, Guangzhou, China

**Keywords:** functional near-infrared spectroscopy (fNIRS), NIVR-Exergames, resistance exercise, cognition, motor, neural response mechanism of cerebral cortex, 2-back

## Abstract

**Background:**

Exercise is one of the effective ways to improve cognition. Different forms of exercises, such as aerobic exercise, resistance exercise, and coordination exercise, have different effects on the improvement of cognitive impairment. In recent years, exergames based on Non-Immersive Virtual Reality (NIVR-Exergames) have been widely used in entertainment and have gradually been applied to clinical rehabilitation. However, the mechanism of NIVR-Exergames on improving motor cognition has not been clarified. Therefore, the aim of this study is to find whether NIVR-Exergames result in a better neural response mechanism to improve the area of the cerebral cortex related to motor cognition under functional near-infrared spectroscopy (fNIRS) dynamic monitoring in comparison with resistance exercise (resistance band stretching).

**Methods:**

A cross-over study design was adopted in this study, and 15 healthy young subjects (18–24 years old) were randomly divided into group A (*n* = 8) and group B (*n* = 7) according to a computerized digital table method. Task 1 was an NIVR-Exergame task, and Task 2 was resistance band stretching. Group A first performed Task 1, rested for 30 min (i.e., a washout period), and then performed Task 2. Group B had the reverse order. The fNIRS test was synchronized in real time during exercise tasks, and heart rate measurements, blood pressure measurements, and 2-back task synchronization fNIRS tests were performed at baseline, Post-task 1, and Post-task 2. The primary outcomes were beta values from the general linear model (GLM) in different regions of interest (ROIs), and the secondary outcomes were heart rate, blood pressure, reaction time of 2-back, and accuracy rate of 2-back.

**Results:**

The activation differences of Task 1 and Task 2 in the right premotor cortex (PMC) (*P* = 0.025) and the left PMC (*P* = 0.011) were statistically significant. There were statistically significant differences in the activation of the right supplementary motor area (SMA) (*P* = 0.007), left dorsolateral prefrontal cortex (DLPFC) (*P* = 0.031), left and right PMC (*P* = 0.005; *P* = 0.002) between baseline and Post-task 1. The differences in systolic pressure (SBP) between the two groups at three time points among women were statistically significant (*P*1 = 0.009, *P*2 < 0.001, *P*3 = 0.044).

**Conclusion:**

In this study, we found that NIVR-Exergames combined with motor and challenging cognitive tasks can promote the activation of SMA, PMC and DLPFC in healthy young people compared with resistance exercise alone, providing compelling preliminary evidence of the power for the rehabilitation of motor and cognitive function in patients with central nervous system diseases.

## Introduction

The incidence of cognitive dysfunction gradually increases with age (Feigin et al., 2009; Reitz et al., 2011, 2020). The prevalence of mild cognitive dysfunction for those over 65 years old is 10–20% (Langa and Levine, 2014), and it significantly decreases the ability to live independently, which increases burdens on families and society. Therefore, it is particularly important to find how to effectively prevent dysfunction.

In a review of cognitive-enhancing technologies, certain drugs (nootropic, methylphenidate, and modafinil) can enhance cognitive abilities, including attention, concentration, and vigilance. But it can also be dangerous, causing headaches, diarrhea, insomnia, fatigue, tremors and nausea. Among non-pharmaceutical interventions, both exercise and music are one of the effective methods to improve cognition (Al-Shargie et al., 2019; Wu et al., 2019). Different types of music have different effects on cognition and perception. For example, fast-paced music can improve a person’s concentration and cognitive function, while maintaining vigilance. Moreover, many studies have confirmed that exercise can promote neuroplasticity of the brain, thereby improving cognitive function. The potential related mechanisms include the improvement of oxygen consumption and increased cerebral blood flow, which promote brain-cell regeneration in brain regions related to cognitive function (Han et al., 2017). Exercise could also up-regulate brain-derived neurotrophic factor (BDNF) and downstream redox signaling pathways, enhance the repair process of antioxidant and oxidative damage, and increase brain metabolism and neuronal activation (Quan et al., 2020). In addition, exercise could also regulate the release of neurotransmitters (Cassilhas et al., 2016), affect the neurotrophic effect of BDNF (Vaynman and Gomez-Pinilla, 2005), and promote the generation of new neurons (van Praag et al., 2005), thereby promoting neural plasticity.

Different forms of exercise, such as aerobic exercise, resistance exercise, and coordination exercise, have different effects on cognitive dysfunction (Hötting and Röder, 2013). There are relevant behavioral data suggesting that coordinated exercise has greater benefits over pure aerobic exercise in different exercise types. Voelcker-Rehage et al. (2011) conducted a 12-month longitudinal study and showed that cardiorespiratory training such as aerobic exercise was associated with increased sensorimotor network activation and had a positive impact on control tasks (Voelcker-Rehage et al., 2011). Furthermore, coordination exercise was associated with increased visual-spatial network activation and had a positive effect on cognitive processing speed, but the specific improvement mechanism remained unclear. Meanwhile, the neural regulation mechanism of resistance exercise in related brain regions remains to be studied. Therefore, it is important to clarify the influence of different forms of exercise on brain function, which could be helpful to guide clinical cognitive training and improve treatment efficiency.

Related research indicates that traditional action video games (such as “Jumping Square”) usually involve visual spatial perception, working memory, executive function, and information processing speed. Repeated game training could shorten reaction time without reducing accuracy or continuing attention (Dye et al., 2009; Bediou et al., 2018). A meta-analysis by Bediou et al. (2018) demonstrated that traditional action video games could significantly enhance top-down attention and spatial perception. A review by Al-Shargie et al. (2019) has found particularly positive effects of Action Video Game on perception, sustained attention, memory, cognitive skills, and decision-making. Such skills include sustained attention and preservation of multitasking tendencies. In addition, video games are often accompanied by rhythmic music, and may have superimposed effects on cognitive improvement. Nevertheless, there are still some drawbacks in traditional action video games. For example, most traditional video games, such as action, adventure, imitation, and strategy games, only involve using the fingers to control the game interface (Straker et al., 2014). Thus, they mainly exercise the abilities of reaction and hand-eye coordination to improve cognitive function, but they rarely involve large-scale activities of the whole body. Overuse of a mouse or keyboard to play games could lead to health problems such as tenosynovitis and benign joint hypermobility syndrome (Queiroz et al., 2018). In addition, prolonged sitting may lead to adverse health consequences such as musculoskeletal pain or syndromes (de Rezende et al., 2014; Straker et al., 2014; Queiroz et al., 2018). Therefore, a healthier, efficient, and feasible way of games to improve cognitive function is needed.

In recent years, with the continuous development of video games, exergames based on Non-Immersive Virtual Reality (NIVR-Exergames) have been widely used in entertainment and have gradually been applied to clinical rehabilitation. Clinical studies have shown that sports games may be beneficial to the improvement of motor function and cognitive function (Swinnen et al., 2020). One example is the game “Just Dance” on the Nintendo Switch. The player mainly uses a visual display screen and a controller to complete dance movements through body movements that follow the rhythm of music (Adcock et al., 2022), and the game provides real-time feedback.

In clinical rehabilitation, the advantages of such games are embodied in various aspects. Patients perform active and systematic repetitive training through specific game tasks involving different cognitive abilities, which realize the reorganization of neural circuits and improve the treatment effect (Gamito et al., 2017). Furthermore, through human machine interaction technology, dynamic feedback and incentive stimulation can be provided, and the actions of patients can be corrected immediately, thereby improving the enthusiasm for training (Gamito et al., 2017). Another benefit is that extensive visual and auditory stimulation as an external factor in training (Pompeu et al., 2012) provides a more attractive, novel, and rich training environment than conventional rehabilitation therapy (Mirelman et al., 2016), thereby increasing patient compliance during long-term rehabilitation (Pompeu et al., 2012).

Non-Immersive Virtual Reality-Exergames combine the cognitive training provided by traditional video games with a form of exercise training (Pichierri et al., 2011). However, further research is needed to determine whether they can integrate the advantages of direct cognitive improvement (game elements improving cognition through cognitive training) and indirect cognitive improvement (exercise elements improving cognition). The results could lead to the realization of a double superposition effect of NIVR-Exergames on cognitive function improvement.

Functional near-infrared spectroscopy (fNIRS) is an optical and non-invasive neuroimaging technology that has been widely used in the assessment of cognitive-related brain functions. Examples include a study on the effect of caffeine on cognitive function using the Stroop task (Yuan et al., 2020) and a study on the change of fall-risk-related brain functional connectivity when using smart phones while walking (Takeuchi et al., 2016). fNIRS is sensitive to mental task load and practice level. An article provide evidence of the fNIRS deployment in the field for its ability to monitor hemodynamic changes that are associated with relative cognitive workload changes of operators. fNIRS allows real-time assessment and has the advantages of portability, economy, and non-invasiveness compared with other brain functional imaging technologies, such as electroencephalography (EEG), magnetoencephalography (MEG), and functional magnetic resonance imaging (fMRI) (McKendrick et al., 2016; Curtin et al., 2019; Pinti et al., 2020). These advantages are especially apparent for subjects with high exercise tolerance (Liu et al., 2016). Furthermore, the method is suitable for subjects performing physical exercise tasks.

Studies have shown that simple exercise and NIVR-Exergames can improve cognitive function (Sakudo, 2016). However, most of the related studies have focused on the function and neuroimaging effects before and after treatment (Saposnik et al., 2016; Bonilauri et al., 2020), and there has been a lack of real-time dynamic research on neural regulation mechanisms.Therefore, the aim of this study is to explore whether NIVR-Exergame tasks lead to a better neural response mechanism to improve cerebral cortex areas related to motor cognition. This was accomplished based on fNIRS for real-time monitoring of brain activation while playing NIVR-Exergames (Nintendo’s “Just Dance”). The results were compared to those obtained with stretching using a resistance band. The findings could guide clinical treatment and home rehabilitation for cognitive dysfunction in the future.

## Materials and Methods

### Subjects

This study recruited 15 healthy young subjects (8 males and 7 females) from the Fifth Affiliated Hospital of Guangzhou Medical University. The inclusion criteria were the following: (1) healthy young people between 18 and 24 years old; (2) right handedness according to the Edinburgh Handedness Inventory;voluntarily signing of an informed consent form. The exclusion criteria were: (1) a history of diseases with motor dysfunction, such as fracture, limb pain, etc.; (2) cardiopulmonary diseases resulting in inability to complete the exercise; (3) sudden diseases occurring during the task affecting the experimental results; (4) history of brain-related diseases with cognitive dysfunction or medications that improve cognitive function being taken; (5) severe mental disorders; and (6) no relevant professional training.

### Statements of Ethical Consideration

This study was performed in accordance with the principles of the Declaration of Helsinki and was approved by the Ethics Committee of the Fifth Affiliated Hospital of Guangzhou Medical University (Ethics No.: GYWY-L2021 - 70). The intervention program has also been registered with the International Clinical Laboratory Registry (registration number: ChiCTR2100054358). All subjects signed an informed consent form before participating.

### Study Design

**FIGURE 1:**
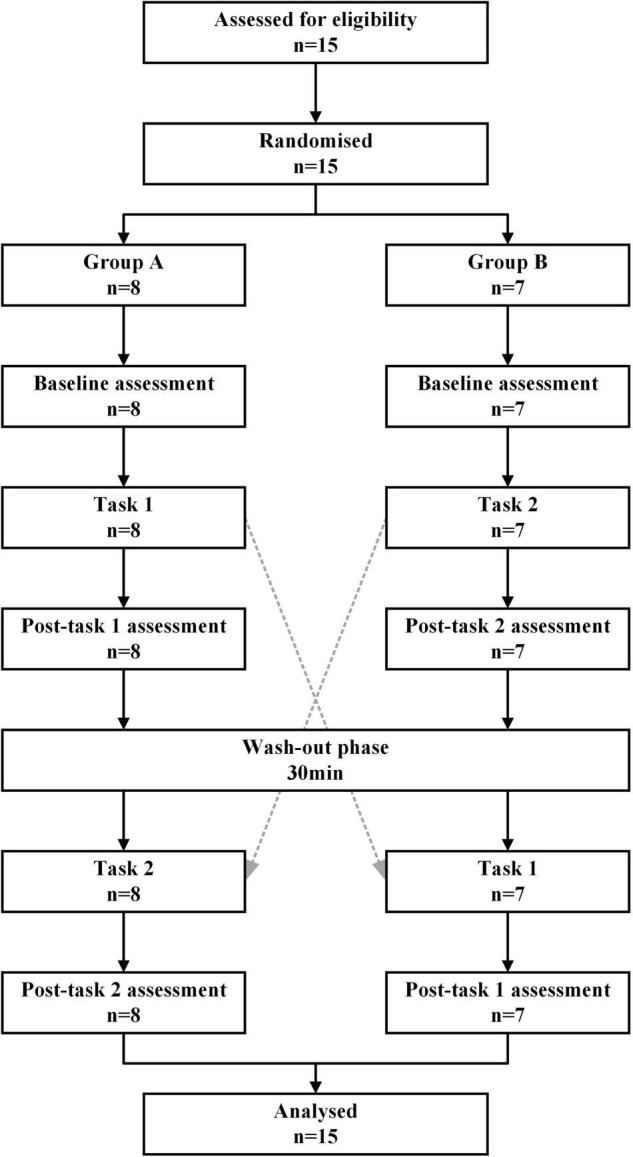
Cross-over design of the study. Task 1: NIVR-Exergame task; Task 2: resistance band stretching task; Post-task 1: after the NIVR-Exergame task; Post-task 2: after the resistance band stretching task. min, minute.

A crossover study design was adopted in this study (Figure 1), and the subjects were randomly divided into group A (*n* = 8) and group B (*n* = 7) according to the computerized digital table method with a ratio of 1:1. The experimental scheme consists of two tasks and three measurement time points (baseline, Post-task 1, and Post-task 2). An NIVR-Exergame, “Just Dance” by Nintendo, was used in Task 1, and resistance band stretching was used in Task 2. Group A first performed Task 1, rested for 30 min (i.e., a washout period), and then did Task 2. Group B had the opposite order. Heart rate measurements, blood pressure measurements, and the 2-back test were performed at baseline, Post-task 1, and Post-task 2. The fNIRS measurement was carried out simultaneously during the two exercise tasks and 2-back task.

### Motor Exercise Tasks

#### Resistance Band Stretching Task

The subjects used a conventional red resistance band (Thera-Band, United States) to perform Task 2. The subjects were required to complete the stretching of the upper limbs as much as possible within 3 min and to keep the position of the feet unchanged.

### NIVR-Exergame Task

Participants used the game “Just Dance” (Soy Yo-Snake Version) based on the Nintendo Switch system (Nintendo Co., Ltd., Kyoto, Japan) for Task 1. Subjects were asked to wear a controller on their right hand and to play the “snake” role. The NIVR-Exergame task lasted for 3 min. During the task, only the upper limbs of subjects were required to complete the action with the requirements of the game and try to keep the position of the feet unchanged (this was done to take into account the stretching task of the upper limb with the resistance band).

### Clinical Assessment

Heart rate and blood pressure were measured at baseline, Post-task 1, and Post-task 2 using an upper-arm electronic sphygmomanometer (Model: U10L, OMRON Healthcare Co., Ltd., China), which met the standards of the American Medical Device Promotion Association (AAMI). Heart rate was recorded in beats per minute, and blood pressure was recorded in mmHg. Blood pressure and heart rate were recorded immediately after the exercise stopped. Subjects were asked to keep the body straight in a sitting position, and the center of the cuff was kept at the same height as the heart. Blood pressure and heart rate were measured three times in total, and all measurements were collected by a well-trained researcher.

### Cognitive Assessment

**FIGURE 2:**
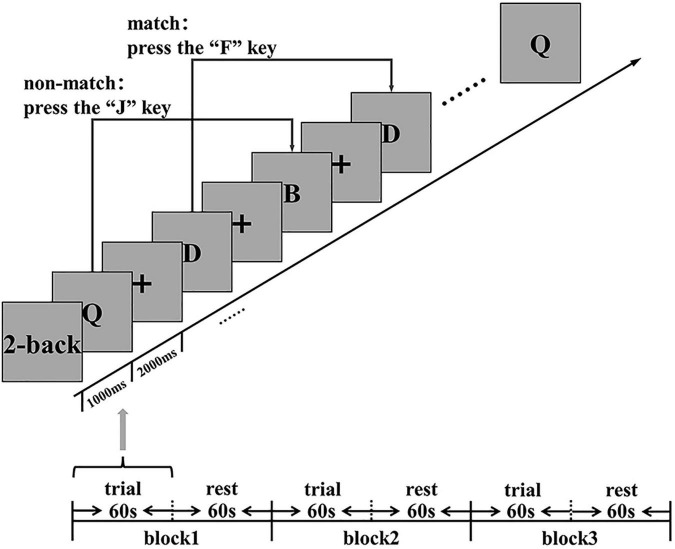
Cognitive function (working memory) assessment: 2-back stimulus display. ms, millisecond; s, second.

This study used the N-back task (2-back) based on E-prime (Psychological Software Tools Company) to evaluate the cognitive function (working memory) of subjects at three time points (baseline, Post-task 1, and Post-task 2) (Figure 2). Before the 2-back task, the subjects were asked to practice for 2 min first to eliminate the influence of the familiarity effect on the subsequent evaluation. The 2-back task paradigm continuously presents a series of random letters (A-H) in the center of the display on a laptop running Windows. Subjects have to remember the letters that appear and respond as quickly and accurately as possible by clicking a response button corresponding to the computer keyboard.

In the 2-back task, the subjects need to compare the letters that they see at present with the letters at an interval ahead of them. The subjects are asked to press “F” (if the two letters are the same), or “J” (otherwise). This task consists of three continuous 2-back blocks. A separate block consists of 60 s of trial and 60 s of rest. The trial presentation time is 1000 ms, and the reaction time is 2000 ms, which makes a total time of 3000 ms. The subjects received no feedback on their scores during the test. During the evaluation task, fNIRS was used to synchronously collect hemodynamic signals in the cortex. The main measurement indexes are the number of correct answers, the number of wrong answers, the number of missing answers, the accuracy rate (AR) {(the number of correct answers/total answers) × 100%}, and the average reaction time (RT).

### Functional Near-Infrared Spectroscopy Assessment/Setting

#### Functional Near-Infrared Spectroscopy

**FIGURE 3:**
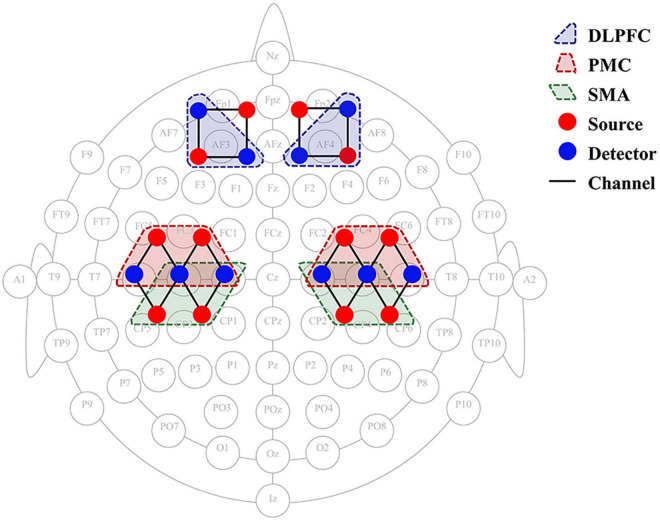
fNIRS 24-channel placement. SMA, supplementary motor cortex; PMC, premotor cortex; DLPFC, dorsolateral prefrontal cortex; fNIRS, functional near-infrared spectroscopy.

Hemodynamic changes were monitored in real time during the exercise tasks and 2-back task using a two wavelengths (730 and 850 nm) CW-fNIRS equipment (Nirsmart, Danyang Huichuang Medical Equipment Co., Ltd., China). 12 emitters and 10 detectors constituting 24 channels were placed in accordance with the international 10–20 system (Figure 3), using a fixed 3cm source-detector spacing. The cerebral cortex hemodynamic response was collected and recorded at a sampling rate of 10 Hz.

#### Regions of Interest

The statistical parameters of the Montreal Neurology Institute (MNI) were mapped to NIRS_SPM software to calculate the coordinates of each signal source and detector position. The software provides the Brodmann region corresponding to each fNIRS channel. Based on the Brodmann partition, six regions of interest (ROIs) were identified in the bilateral dorsolateral prefrontal cortex (DLPFC), supplementary motor areas (SMA), and premotor cortex (PMC) (Figure 3). The channel distribution of the selected ROI is such that the left DLPFC corresponds to channels 5 and 6, and the right DLPFC corresponds to channels 1 and 2. The left and right SMAs correspond to channels 17 and channel 12, respectively. The left PMC corresponds to channels 21, 22, and 23, and the right PMC corresponds to channels 14, 15, and 16, respectively. The changes in oxyhemoglobin concentrations in the ROIs were collected during the 2 motor tasks and at the 3 time points, and the beta values were calculated using the general linear model (GLM) to quantify the correlation between brain regions and tasks.

#### Functional Near-Infrared Spectroscopy Data Processing and Analysis

All the original optical intensity time series were derived from NIRSMART. Firstly, the channels with low-quality signals were removed to reduce the error according to the received optical signal quality of each channel. Secondly, the modified Beer-Lambert law (Herold et al., 2017) is used to convert the optical intensity into relative change of oxyhemoglobin (HbO2) and deoxyhemoglobin (HbR) concentrations. Then, the NIRS-SPM toolbox^1^ (Dye et al., 2009) was used to process the HbO2 signal of the target channel, since HbO2 generally has better signal-noise ratio than the HbR signal (Strangman et al., 2002). A statistical parameter mapping method based on the GLM is used to fit the fNIRS data using NIRS-SPM. For each separate HbO2 time series during three different task (NIVR-Exergame Task, Resistance Band Stretching Task, 2-back Task), the model parameters for GLM could be described as y∼1 + x_i_ in Wilkinson-notation, where y represents the hemodynamic response, x_i_ denotes the corresponding regressor for each task (de Winkel et al., 2017). Because several physiological processes (e.g., respiration, blood-pressure changes, heartbeat, etc.) are known to produce structured “noise” within the data (i.e., autocorrelation), a precoloring treatment is performed, through which such temporal correlations are “swamped” by an imposed 4s Gaussian temporal correlation structure and are thus effectively reduced. Wavelet minimum description length detrending algorithm is applied to the both model and data to count for the global trends. Then model estimation was done and β value was used as the indicator of brain activation. The largest beta outcome for all channels within a single ROI is selected and entered into the group-level analyses reported below (Ashlesh et al., 2020; Baker et al., 2020). Finally, the activation beta value (the weight coefficient in the GLM) in each task is obtained to quantify the correlation between the brain region and the task.

### Outcomes

The primary outcome indicators are the GLM beta values in different ROIs. The secondary outcome indicators are the heart rate, blood pressure, reaction time of 2-back, and accuracy rate of 2-back.

### Sample Size

The sample size for the comparison of the two exercise interventions (using a paired *t*-test) was estimated using G*Power 3.0 (Faul et al., 2007). The sample size was calculated using data regarding cortical activation of the premotor cortex from five subjects in a preliminary experiment. The analysis indicated that the total sample size should be 12 based on a level of significance of 0.05 and statistical power of 0.8.

### Statistical Analysis

SPSS 25.0 was used for statistical analysis of the data. When processing the clinical evaluation data, the influence of gender on the results was tested by comparison between male and female groups using an independent-samples *t*-test. Multicomparison correction procedure was ued to avoid the type I error. An analysis of variance (ANOVA) was used to compare the effects of three different time points on the results in the male and female groups. When processing cognitive assessment data, a paired t-test was used to compare the effects of the two different tasks on the results. A repeated measure ANOVA was used to analyze the effect of multiple factors. *P* < 0.05 indicated that the difference was statistically significant.

## Results

The study recruited 15 healthy subjects (8 males and 7 females) between 18 and 24 years old.

Baseline characteristics show the data of male and female group, including age, height, weight, body mass index (BMI), resting blood pressure, and heart rate (HR) (Table 1). Compared with the female group, the male group had greater height (171.938 ± 5.710 cm in the male group and 160.143 ± 4.811 cm in the female group, *P* = 0.001), weight (60.913 ± 6.132 kg in the male group and 46.686 ± 3.231 kg in the female group, *P* < 0.001), BMI (20.633 ± 2.158 in the male group and 18.210 ± 1.069 in the female group, *P* = 0.019), baseline systolic pressure (SBP) (124.625 ± 9.039 mmHg in the male group and 111.571 ± 5.884 mmHg in the female group, *P* = 0.006). These differences between groups were statistically significant.

**TABLE 1:** Baseline information.

	Male group (*n* = 8)	Female group (*n* = 7)	*P*-value
Age (year)	21.000 ± 0.756	21.143 ± 1.952	0.851
Height (cm)	171.938 ± 5.710	160.143 ± 4.811	0.001**
Weight (kg)	60.913 ± 6.132	46.686 ± 3.231	<0.001***
BMI	20.633 ± 2.158	18.210 ± 1.069	0.019*
**Cardiovascular response**			
SBP (mmHg)	124.625 ± 9.039	111.571 ± 5.884	0.006**
DBP (mmHg)	78.750 ± 6.585	75.134 ± 5.113	0.263
HR (beat/min)	75.134 ± 5.113	79.000 ± 9.055	0.197

Significance level set at P < 0.05. Significant correlations are marked with *P < 0.05, **P < 0.01, and ***P < 0.001. BMI, body mass index; SBP, systolic blood pressure; DBP, diastolic blood pressure; cm, centimeter; kg, kilogram; mmHg, millimeters of mercury; min, minute; P, P value.

The results of the ANOVA with a 2 × 3 factorial design shows the effects of gender and time on cardiovascular response and whether there is interaction between them (Table 2). The results show that the differences in SBP measured for different genders and at different time points were statistically significant (gender factor: F = 31.137, *P* < 0.001; time factor: F = 11.745, *P* < 0.001). However, the interaction between gender and time was not statistically significant (F = 3.665, *P* = 0.581).

**TABLE 2:** Results of 2 × 3 factorial analysis design on gender and time for HR, SBP, DBP during Baseline, Post-task 1 and Post-task 2.

	Main effect (Gender)		Main effect (Time)		Interaction effect	
	*F*-value	*P*-value	*F*-value	*P*-value	*F*-value	*P*-value
HR (beat/min)	2.120	0.153	0.826	0.445	0.161	0.852
SBP (mmHg)	31.137	<0.001***	11.745	<0.001***	0.540	0.587
DBP (mmHg)	1.795	0.188	3.091	0.057	0.339	0.714

Significance level set at P < 0.05. Significant correlations are marked with *P < 0.05, **P < 0.01, and ***P < 0.001. HR, heart rate; SBP, systolic blood pressure; DBP, diastolic blood pressure; min, minute; mmHg, millimeters of mercury; P, P value. Significant correlations are marked with ***P < 0.001.

**FIGURE 4:**
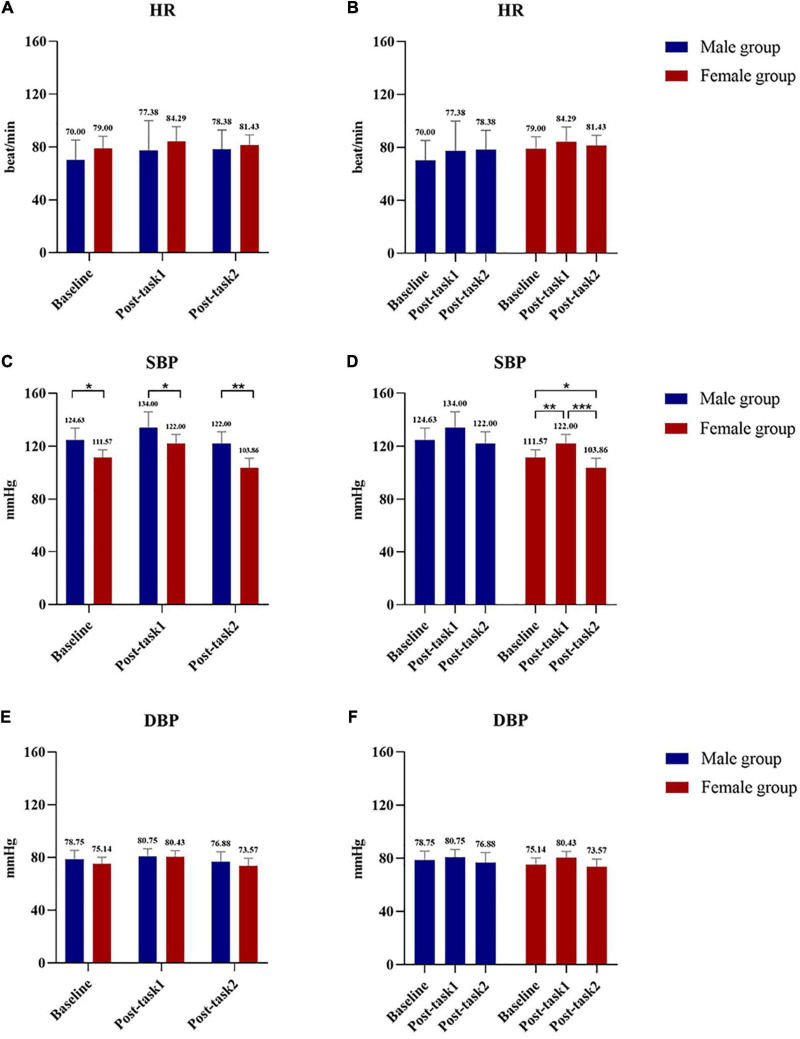
Comparison of HR, SBP and DBP between male group and female group at 3 time points between groups and within groups. **(A)** Comparison of HR at Baseline, Post-task 1 and Post-task 2 between male and female group. **(B)** Comparison of HR at Baseline, Post-task 1 and Post-task 2 in male group and female group. **(C)** Comparison of SBP at Baseline, Post-task 1 and Post-task 2 between male and female group. **(D)** Comparison of SBP at Baseline, Post-task 1 and Post-task 2 in male group and female group. **(E)** Comparison of DBP at Baseline, Post-task 1 and Post-task 2 between male and female group. **(F)** Comparison of DBP at Baseline, Post-task 1 and Post-task 2 in male group and female group. Post-task 1: after the NIVR-Exergame task; Post-task 2: after the resistance band stretching task. mmHg, millimeters of mercury; min, minute; HR, heart rate; SBP, systolic blood pressure; DBP, diastolic blood pressure.

The post-test results shows that SBP in the male group and female group showed statistically significant differences at baseline (male group: 124.625 ± 9.039 mmHg; female group: 111.571 ± 5.884 mmHg, *P* = 0.006), Post-task 1 (male group: 134.000 ± 11.916 mmHg; female group: 122.000 ± 6.928 mmHg, *P* = 0.036), and Post-task 2 (male group: 122.000 ± 8.816 mmHg, female group: 103.857 ± 7.105 mmHg, *P* = 0.001) (Figure 4). SBP was compared at 3 time points in the female group, and the results showed that the differences between the two groups were statistically significant (baseline: 111.571 ± 5.884 mmHg vs. Post-task 1: 122.000 ± 6.928 mmHg, *P* = 0.009; baseline: 111.571 ± 5.884 mmHg vs. Post-task 2: 103.857 ± 7.105 mmHg, *P* < 0.001; Post-task 1: 122.000 ± 6.928 mmHg vs. Post-task 2: 103.857 ± 7.105 mmHg, *P* = 0.044).

**FIGURE 5:**
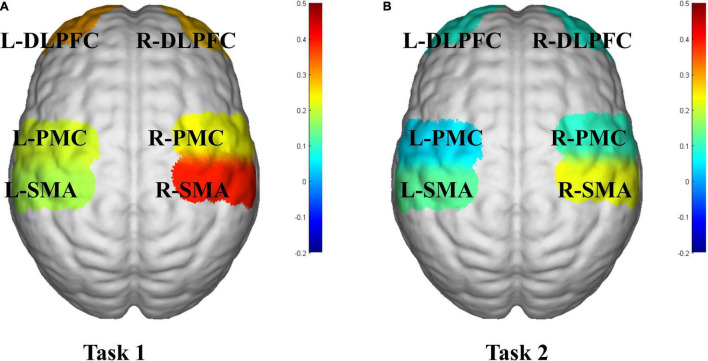
fNIRS activation maps of two motor tasks. **(A)** Task 1, NIVR-Exergame task; **(B)** Task 2, resistance band stretching task. The beta values are indicated by color. SMA, supplementary motor cortex; PMC, premotor cortex; DLPFC, dorsolateral prefrontal cortex; L, left; R, right.

**FIGURE 6:**
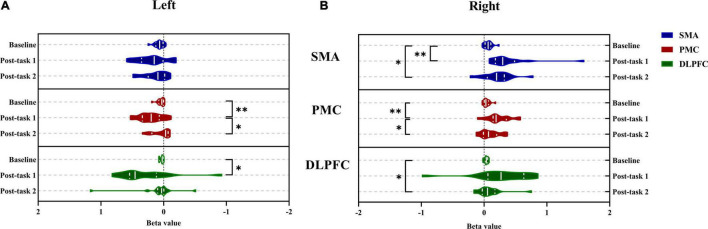
Comparison between the left and right brain of bate value in different regions of interest during Baseline, Post-task 1 and Post-task 2. **(A)** The left brain; **(B)** the right brain. *P < 0.05 and ^**^P < 0.01. SMA, supplementary motor cortex; PMC, premotor cortex; DLPFC, dorsolateral prefrontal cortex; Post-task 1: after the NIVR-Exergame task; Post-task 2, after the resistance band stretching task.

The activation of the ROIs during Task 1 and Task 2 is also shown according to the GLM (Table 3). In Task 1, the beta values of the right PMC and left PMC were higher than those in Task 2 (right PMC: Task 1: 0.229 ± 0.186 vs. Task 2: 0.096 ± 0.156, *P* = 0.025; left PMC: Task 1: 0.206 ± 0.188 vs. Task 2: 0.043 ± 0.144, *P* = 0.011) (Figure 5). The activation of the right SMA, right PMC, left PMC, right DLPFC and left DLPFC regions of the brain was statistically significant when time was the main effect (F = 5.870, *P* = 0.007, F = 7.894, *P* = 0.002; F = 7.550, *P* = 0.002; F = 5.305, *P* = 0.021; F = 3.199, *P* = 0.056) (Table 4). The results of *post hoc* tests for comparison of the GLM beta values in different ROIs in the left and right brain regions and at different time points are shown (Figure 6). Compared with baseline, Right SMA (*P* = 0.007), Right and Left PMC (*P* = 0.002; *P* = 0.005) and Left DLPFC (*P* = 0.031) were significantly activated at Post-task 1; Right SMA and Right DLPFC (*P* = 0.037; *P* = 0.013) were significantly activated at Post-task 2. Compared with Post-task 1, Right and Left PMC (*P* = 0.025; *P* = 0.011) were significantly activated at Post-task 2.

**TABLE 3:** The comparison of beta value based on GLM analysis between Task 1 and Task 2 using Paired-Samples T test.

ROIs	Task 1	Task 2	T-value	*P*-value
**SMA**				
Right SMA	0.390 ± 0.371	0.222 ± 0.234	1.540	0.146
Left SMA	0.182 ± 0.245	0.126 ± 0.187	0.895	0.386
**PMC**				
Right PMC	0.229 ± 0.186	0.096 ± 0.156	2.502	0.025*
Left PMC	0.206 ± 0.188	0.043 ± 0.144	2.949	0.011*
**DLPFC**				
Right DLPFC	0.274 ± 0.452	0.083 ± 0.214	1.554	0.143
Left DLPFC	0.296 ± 0.430	0.083 ± 0.342	1.631	0.125

Significance level set at P < 0.05. Significant correlations are marked with *P < 0.05, **P < 0.01, and ***P < 0.001. ROIs, regions of interest; SMA, supplementary motor cortex; PMC, premotor cortex; DLPFC, dorsolateral prefrontal cortex; Task 1, NIVR-Exergame task; Task 2, resistance band stretching task; GLM, general linear model; T, T value; P, P value.

**TABLE 4:** The result of repeated measure ANOVA for the beta values of ROIs during Baseline, Post-task 1 and Post-task 2.

ROIs	*F*-value	*P*-value
**SMA**		
Right SMA	5.870	0.007**
Left SMA	1.719	0.198
**PMC**		
Right PMC	7.894	0.002**
Left PMC	7.550	0.002**
**DLPFC**		
Right DLPFC	5.305	0.021*
Left DLPFC	3.119	0.056*

Significance level set at P < 0.05. Significant correlations are marked with *P < 0.05, **P < 0.01, and ***P < 0.001. ROIs, regions of interest; SMA, supplementary motor cortex; PMC, premotor cortex; DLPFC, dorsolateral prefrontal cortex; Post-task 1, after the NIVR-Exergame task; Post-task 2, after the resistance band stretching task; F, F value; P, P value.

## Discussion

In recent years, NIVR-Exergames have effectively integrated the advantages of sports and traditional games. As a commercial mass-entertainment product, NIVR-Exergames have also been gradually applied in the field of clinical rehabilitation (Swinnen et al., 2020). However, there are few studies on NIVR-Exergames to improve motor cognition, and the relevant mechanisms are not yet clear. In this study, NIVR-Exergames and a resistance-band stretching exercise were compared, and fNIRS was used for the first time to monitor the activation of ROIs during and after exercise. Furthermore, the neural regulation mechanism of NIVR-Exergames in improving motor cognition was explored. The results show that the NIVR-Exergame task was more helpful for the immediate response of ROIs than the resistance-band stretching task and was not affected by gender. However, the SBP after two exercise tasks was affected by gender factors.

Compared with the resistance-band stretching task, the NIVR-Exergame task significantly activated the bilateral PMC. Its mechanism may be related to the PMC’s participation in motion control, motor planning, and learning (Kantak et al., 2012). The PMC is located in the frontal lobe and the front end of the anterior central gyrus adjacent to the posterior primary motor cortex and is a functional area related to movement in the frontal lobe of the brain (Nakajima et al., 2019; Fornia et al., 2020). Studies have shown that brain activities in motor planning and various cognitive tasks are jointly located in the PMC (Hanakawa, 2011).

This study used NIVR-Exergames combined with dance elements for training. Studies have shown that dance movement therapy (DMT) has obvious benefits in improving cardiovascular health (Li et al., 2015), musculoskeletal health (Matthews et al., 2006), dexterity, balance, and overall endurance. The training process involves the practice of sensory motor skills, which requires observation of specific movements and imitation, learning, and motor planning (Mura et al., 2018), which are closely related to PMC function. At the same time, when subjects participated in interactive virtual tasks and imitate the interactive process, the PMC performs predictive decoding of virtual tasks, action observation, and prediction, and then promotes action imitation (Sacheli et al., 2019).

The resistance-band stretching task involves a relatively simple, repetitive, mechanized upper-limb movement that is less affected by rhythm perception, motor planning, and learning factors. This explains why the NIVR-Exergames significantly activated the bilateral PMC compared with the resistance-band stretching task in this study. Studies have shown that the execution and imagination of motion require activation of the connectivity of the brain region, and the common coupling nodes include DLPFC-PMC and PMC-SMA (Kim et al., 2018). We speculate that the activation of PMC in the NIVR-Exergame task may be the key connection point of brain activation during cognitive-motor tasks and that it plays an important role in improving motor and cognitive function.

The results of this experiment showed that the NIVR-Exergame task and resistance-band stretching task could significantly activate the right SMA compared with the baseline. The potential mechanism may be related to the specialization in the right hemisphere region The SMA is located in the precentral gyrus of the frontal lobe and plays a key role in the execution of autonomous motion. It is not only involved in motor execution, but also movement sequence organization (Gerloff et al., 1998). Studies have shown that performing dexterous exercises involves spatial coordination of multi-joint, proximal, and distal muscle activity. This requires complex movement sequences that can be acquired and refined through extensive exercises, thus allowing engagement in skilled activities such as typing, dancing, musical performance, and exercise (Strick et al., 2021). Therefore, improving the dexterity of movement is potentially associated with SMA activation.

In addition, previous studies have found significant functional specialization in music perception, and the central areas of perception of pitch, melody, and harmony are located in the right hemisphere (Tramo, 2001; Tramo et al., 2002). The right hemisphere is the center of visualization, imagination, and conceptualization, with advantages in metaphorical thinking, gameplay, and finding solutions. The NIVR-Exergame task in this study requires (Hoppe, 1988) subjects to complete a series of links such as music rhythm perception, game video recognition, motion imagination, and imitation. The resistance-band stretching task in this study also requires subjects to exercise in rhythm. The functions of these links are closely related to the specialization of music perception areas. Therefore, the significant activation of the right SMA may be dominated by music-related right-hemisphere specialization.

The results show that NIVR-Exergames have more development potential than resistance exercise and could be preferable as a method of clinical and home rehabilitation training. In clinical rehabilitation, targeted training such as music and games is of great significance for patients with brain injury, especially right hemisphere injury. Compared with traditional exercise rehabilitation, it has obvious advantages in restoring and improving the dysfunction of patients.

Moreover, the results of this experiment also show the activation of DLPFC by the NIVR-Exergame task. DLPFC is related to cognitive, emotional, and sensory processing and is a key node in multiple brain networks (Seminowicz and Moayedi, 2017). Studies have shown that the DLPFC, especially the tail, combines spatial information with object identity, behavior rules, reward mechanisms, and other information and plays a key role in the cognitive control of motor behavior (Hoshi, 2006). The cognitive function assessment results of the 2-back task synchronous fNIRS test showed that there was significant difference in the Left DLPFC activation between Post-task 1 and Post-task 2. The results suggest that NIVR-Exergames may play an important role in maintaining and improving cognitive function.

However, the 2-back results show that there was no significant difference in RT and AR between Baseline and Post-task 1. Previous studies have found that the improvement of working memory function after a single aerobic exercise depends on the baseline working memory function but is irrelevant to exercise intensity (Yamazaki et al., 2018). That is, subjects with better performance in baseline assessment are less affected by exercise factors, while subjects with poor performance are greatly affected by exercise factors. Considering that the subjects in this study were healthy and young, their baseline performance was good, so the cognitive improvement effect after exercise was not obvious.

In addition, a number of studies have shown that a single short-term exercise has no effect on working memory. When aerobic exercise is performed three times a week for 12 weeks, subjects show benefits in executive function, memory, and complex attention as early as week 6, and continuous benefits are shown in week 12 (Cabral et al., 2019). Combined with the cognitive function evaluation results of the 2-back task synchronous fNIRS test in this study, the 2-back AT and RT Post-task 1 showed no obvious improvement effect. This may have been because the 2-back task designed in this study was less difficult for healthy young people. At the same time, it may be due to the short time of the single exercise task as well. Moreover, it has been demonstrated in the relevant literature that elevated oxygenated hemoglobin levels of PFC (prefrontal cortex, PFC) in physical exercise and cognitive tasks may be associated with an increase in neurometabolic activation, which is one of the candidate mechanisms for cognitive function improvement (Bediz et al., 2016). In a follow-up study, we could extend the treatment cycle and improve the difficulty of cognitive assessment tasks to further examine the effect of two kinds of exercise tasks on improving motor cognition at the functional level.

In terms of cardiovascular response, previous studies have shown that gender is one of the factors affecting blood pressure (Cheng et al., 2012). The baseline results of this experiment also showed that the mean resting systolic blood pressure of the male group was higher than that of the female group, which was consistent with previous results, although the systolic blood pressures of the two groups were healthy. This study also found that compared with the baseline, the systolic blood pressure of the female group Post-task 1 was significantly increased, while that Post-task 2 was significantly decreased. The potential mechanism of elevation may be related to the heart and nerve response. Immediately after exercise, the sympathetic nervous system is still in an excited state, and the blood vessels in the skin and internal organs contract, while the cardiac contractility is strengthened, resulting in an increase in systolic blood pressure (Teng et al., 2005).

Studies have shown that a potential mechanism of blood pressure reduction after resistance training may be related to the decrease of peripheral vascular resistance (Lopes et al., 2021) or the improvement of autonomic nerve function (Oliveira-Dantas et al., 2020). At the same time, resistance exercise reduces the inflammatory factors in patients with hypertension, thereby reducing the inflammatory response to damage vascular endothelial cells and slowing the proliferation and migration of endothelial cells, thus lowering blood pressure. In clinical practice, resistance training can increase the effect of antihypertensive drugs on individual muscle strength and reduce resting blood pressure (Polito et al., 2021). Therefore, when guiding hypertensive patients, blood pressure should be closely monitored during exercise training, especially systolic blood pressure changes. In addition, resistance exercise may be an optimal option for exercise therapy for patients with hypertension.

It is also worth noting that the decrease in blood pressure in seconds or minutes after resistance exercise can be attributed to sudden perfusion and transient pressure drop in previously occluded muscle blocks and may result in post-exercise hypotension (PEH) (MacDonald et al., 1999). It is suggested that there is a certain risk of resistance exercise, and blood pressure should be closely monitored during exercise training in healthy and low-blood-pressure groups. However, there was no significant change in blood pressure in male groups after exercise in this study, which might be related to the fact that the exercise load of this study was small for them and that they had strong exercise tolerance.

### Limitation

This study was a cross-sectional study that only discussed the effect of single exercise. A follow-up longitudinal study could be done to examine a long-term intervention to explore the potential mechanism of exercise in improving cognition. Moreover, this study can not completely eliminate the interference factors to the signal, and the short channel separation can be used to further reduce the interference to the signal. In addition, this study examined healthy young people, so future studies could look at more different types of exercises among young people, the elderly, and even stroke and hypertension patients, which could shed more light on the neural regulation mechanisms and effects of exercise in improving cognition.

## Conclusion

NIVR-Exergames use a combination of motor and challenging cognitive tasks based on virtual reality technology, and more and more evidence has shown its prospects for clinical application. In this study, synchronous fNIRS was used to monitor two groups of motor tasks in real time. The results showed that NIVR-Exergames might be conducive to the realization of the motor-cognitive dual function superposition effect, which could have significance for guiding future clinical research.

## Data Availability Statement

The data and trial protocol are available from the corresponding author upon reasonable request (QL; qianglin0925@gzhmu.edu.cn).

## Ethics Statement

The studies involving human participants were reviewed and approved by the Ethics Committee of the Fifth Affiliated Hospital of Guangzhou Medical University. The patients/participants provided their written informed consent to participate in this study. Written informed consent was obtained from the individual(s) for the publication of any potentially identifiable images or data included in this article.

## Author Contributions

QL, AY, and HF designed the study. QL, YZ, and TY drafted the manuscript. YZ, TY, and RD performed data analysis. JL, JZ, TP, BZ, HO, and YJ collected the data. RD, JL, JZ, and TP wrote sections of the manuscript. QL, AY, and HF approved the final version of the manuscript. All authors contributed to manuscript revision, read, and approved the submitted version.

## Conflict of Interest

The authors declare that the research was conducted in the absence of any commercial or financial relationships that could be construed as a potential conflict of interest.

## Publisher’s Note

All claims expressed in this article are solely those of the authors and do not necessarily represent those of their affiliated organizations, or those of the publisher, the editors and the reviewers. Any product that may be evaluated in this article, or claim that may be made by its manufacturer, is not guaranteed or endorsed by the publisher.

## Footnotes

1
https://www.nitrc.org/projects/nirs_spm/
