# Supplementary material for: The Effect of Non-immersive Virtual Reality Exergames Versus Band Stretching on Cardiovascular and Cerebral Hemodynamic Response: A Functional Near-Infrared Spectroscopy Study
Source: Front Hum Neurosci. 2022 Jul 12;16:902757. doi: 10.3389/fnhum.2022.902757 (PMC9314640; doi:10.3389/fnhum.2022.902757)
Supplement: Supplementary file 1 [file Table_1.doc]

**Supplementary Table 1**

**The comparison of 2-back assessment during Baseline, Post-task 1 and Post-task 2.**

|  | **Baseline** | **Post-task 1** | **Post-task 2** | ***P*1** | ***P*2** | ***P*3** | ***P*4** |
| --- | --- | --- | --- | --- | --- | --- | --- |
| AR | 0.922 ± 0.158 | 0.978 ± 0.028 | 0.930 ± 0.130 | 0.392 | 0.210 | 0.866 | 0.276 |
| RT | 1351.63 ± 527.045 | 1079.807 ± 377.038 | 1201.888 ± 471.424 | 0.282 | 0.119 | 0.305 | 0.584 |

Notes: Significance level set at *P* < 0.05. Significant correlations are marked with * ( *P* < 0.05 ), ** ( *P* < 0.01 ) and *** ( *P* < 0.001 ). *P*1 indicated the comparison among Baseline, Post-task 1and Post-task 2 ; *P*2 indicated the comparison between Baseline and Post-task 1; *P*3 indicated the comparison between Baseline and Post-task 2; *P*4 indicated the comparison between Post-task 1 and Post-task 2. Post-task 1: after the NIVR-Exergame task; Post-task 2, after the resistance band stretching task.

Abbreviations: AR, accuracy rate; RT, reaction time.
